# Editorial: Host Microbiomics—Effects of Environmental and Physical Stressors, Interventions, and Pathogens on Composition and Function

**DOI:** 10.3389/fgene.2022.931150

**Published:** 2022-07-01

**Authors:** Sarah C. Pearce, Gregory J. Weber

**Affiliations:** ^1^ National Laboratory for Agriculture and the Environment, USDA-ARS, Ames, IA, United States; ^2^ Functional Foods and Nutritional Interventions Team, Combat Feeding Division, Natick, MA, United States

**Keywords:** microbiomics, genomic, environmental, stressor, pathogen

Mounting evidence in recent years has revealed the importance of the complex community of organisms found in, and on, the human body, known collectively as the microbiome including bacteria, viruses, and fungi. Studies have predominately focused on bacterial flora, especially in the gut, lung, and skin, and have highlighted the importance of the microbiota in homeostasis including immune system modulation, epithelial barrier integrity, nutrient absorption, protection against pathogen infection, and maintaining organ system homeostasis. Disruption of a balanced microbiome (dysbiosis) has been linked with altered host function and disease. High-throughput technologies have revealed insight into the diversity of the microbial community composition; however, they have also laid bare our lack of understanding of the specific functions of these microbes, particularly in health and disease. The aim of this Research Topic was to build a broad collection of original research articles utilizing high throughput methods (e.g., 16S, whole genome, metagenomic, transcriptomic, metabolomic, and proteomic) to identify microbiome composition and link it with functional outputs. Stress-inducing and mitigating approaches, such as hypoxia, cold/heat, pathogen, diet/nutritional supplementation, exercise are of particular interest. Intestinal and pulmonary microbiomes are of particular interest, especially studies linking these microbiomes to other organs (e.g., gut-brain, gut-lung, and gut-skin axes). Current interests also include research related to SARS-CoV-2 infection as emerging evidence is showing that it can affect other organ systems and may impact the microbiome.

This Research Topic consisted of four papers, all of which were original research articles. Covered models included mice and rats, to mites, to potatoes, all utilizing various sequencing methods to evaluate microbial species. Combined, these papers have received nearly 3,000 views to date.

One paper examined the storage of potatoes which is extremely important for the food industry. There have been several problems involving rotting and low quality, thus authors examined two separate potato-producing areas and examined the microbial community structure using high throughput sequencing. This study showed that community composition and microbial diversity was different in the two tested regions and due to storage period as well. Fungi were also analyzed. Takeaways from this study were to provide a theoretical basis for biological control of potato diseases to use for maintenance of long-term storage (Xie et al.).

The next paper evaluated stored product mites. Several insect taxa utilize coprophagy (eating feces) to transmit beneficial microbes. Stored product mites are one of the insect types that do this and are documented carriers of microorganisms. This study examined mite growth in conditions with or without mite feces and evaluated microbial community changes. Authors showed that population density was not affected by the presence of feces in two cultures but was affected in two other cultures. Several microbial taxa were associated with fecal treatment, which were correlated with reduced mite fitness. Overall, authors showed that although coprophagy can be used to gain beneficial bacteria, that it can also have negative effects on some mite populations (Green et al.).

A couple of the studies accepted into this Research Topic used more traditional rodent models when investigating microbiomes, specifically intestinal gut flora. The first of these studies focused on chronic atrophic gastritis (CAG), a risk factor for gastric cancer and is associated with a plethora of other health issues. The authors chose to evaluate a more alternative form of treatment for CAG, electroacupuncture, as opposed to drug-targeted therapy. Interestingly, the authors found an increase in gut microbiota believed to have a beneficial effect on gut health (probiotic), including *Oscillospirales*, *Romboutsia,* and *Christensenellaceae*, in rats treated with electroacupuncture. Further, histopathological analysis of the gastric mucosal glands showed less atrophied gastric glands and mucosal tissues compared to non-treated diseased rats, indicating electroacupuncture may have some alleviating effects of CAG and may be through, in part, promotion of healthy gut microbiota (Huang et al.).

Comparatively, another rodent-based study investigated the role of gut microbiota in chronic constipation, and assessed whether fecal microbiota transplantation from non-constipated mice could shift the gut microbiome to a more eubiotic state. The investigators interrogated the gut microbiome via 16S sequencing and subsequent analyses to determine species diversity and composition, as well as performed histopathological assessments on the colon. A total of 12 microbial genera were found to be altered in constipated mouse feces, and interestingly, is a considerably higher number of reported altered microbiota genera compared to previous studies. The investigators also observed a decrease of the mucosal and muscle later thickness, along with molecular regulators AQP8, C-kit, and 5-HT, linking histopathological molecular changes with alterations in microflora (Choi et al.).

The editors would like to thank all the authors who submitted studies to this Research Topic. This topic has highlighted the variety of research studies that are being performed using high-throughput sequencing methods to answer unique scientific questions. We were pleased to observe these studies linking genetic data to phenotypic/physiological outcomes, which is critically important if we are to gain a more complete understanding to the questions we as scientists pose in our respective fields of research.

